# Novel Trainee-Led Psychological Service in Childhood Cancer Survivorship Clinic: A Process Paper

**DOI:** 10.3390/children13050656

**Published:** 2026-05-07

**Authors:** Stephanie J. Glover, Josh Tiller-Ormord, Kelly Anderson, Jessica Busse, Laura Dorneman, Lori Knowles, Susan Lindemulder, Melinda D. Wu, W. Michael Vanderlind

**Affiliations:** 1 Department of Anesthesiology, Perioperative, & Pain Medicine, School of Medicine, Stanford University, Stanford, CA 94305, USA; sjglover@stanford.edu; 2 Randall Children’s Hospital at Legacy Emanuel Medical Center, Portland, OR 97232, USA; jtillero@lhs.org; 3 Division of Pediatric Hematology and Oncology, School of Medicine, Doernbecher Children’s Hospital, Oregon Health & Science University, Portland, OR 97239, USAlindemul@ohsu.edu (S.L.); wume@ohsu.edu (M.D.W.); 4 Childhood Cancer Survivorship Program, Doernbecher Children’s Hospital, Oregon Health & Science University, Portland, OR 97239, USA; bussej@ohsu.edu (J.B.); dorneman@ohsu.edu (L.D.); knowlesl@ohsu.edu (L.K.); 5 Department of Pediatrics, Institute on Development & Disability, Doernbecher Children’s Hospital, Oregon Health & Science University, Portland, OR 97239, USA

**Keywords:** childhood cancer, survivorship, psychosocial, neurocognitive

## Abstract

**Highlights:**

**What are the main findings?**

- A psychology intern created a novel psychoeducational service line embedded within an existing multidisciplinary childhood cancer survivorship clinic.
- Intern-led psychoeducational services included 20-min visits with families.
- Topics spanned multiple areas, including general coping, anxiety management, improving mood, sleep hygiene, accessing school supports, and parent coaching on responding to challenging behaviors.
- De-identified patient satisfaction data show high rates of patient satisfaction.

**What is the implication of the main finding?**

- This novel service line warrants more research using validated outcome measures and examining long-term benefits.
- The psychoeducational consultation service may help to address an “assessment-to-intervention” gap within survivorship programs, which traditionally focus on screening, triage, and referral placement.
- The inclusion of a psychology intern complements core psychology training competencies and, within our institution, provides families with a no-fee service that does not require live supervision from a licensed provider.

**Abstract:**

**Background/Objectives:** Pediatric hematology and oncology patients are at increased risk for psychosocial and neurocognitive difficulties following treatment. Survivorship programs monitor late effects associated with disease and treatment history, with most programs focusing on screening and referring. Relatively less focus is placed on psychoeducation and intervention. The current paper describes the process of creating a novel psychology trainee-led consultation service embedded within a multidisciplinary survivorship clinic. **Methods:** A psychology intern collaborated with clinic staff and reviewed existing literature to inform the nature of the service. Patients seen in clinic met with the psychology intern for a 20-min visit focused on an area of concern identified during existing neuropsychology and/or social work assessment visits. Topics addressed included coping with stress, anxiety management, improving mood, sleep hygiene, parenting responses to common behavioral issues, and the acquisition of academic support. Pilot patient satisfaction data were collected via a questionnaire at the end of the visit, without any patient-identifiable factors attached to response data. **Results:** Most patients and families (90%) found the service helpful. Moreover, the majority of families (90%) found a 20-min service delivery to be adequate. The most common topic area addressed was anxiety management. **Conclusions:** Results demonstrate high patient satisfaction. Advantages of this service include rapid access to a no-fee support addressing common mental health and neurocognitive sequelae of childhood cancer and the expansion of psychology training opportunities. Future research should evaluate the service using validated outcome measures and examine its long-term effects.

## 1. Introduction

Advances in pediatric oncology have led to five-year survival rates of over 85% in North America, resulting in a rapidly growing population of long-term childhood cancer survivors [1,2]. Although many survivors now live decades beyond treatment completion [3], this success is accompanied by a substantial burden of late effects. Beyond the high probability of developing a chronic health condition by mid-adulthood [4], childhood cancer survivors are at increased risk for long-term psychosocial and neurocognitive difficulties. Early identification and intervention to address psychosocial needs throughout survivorship have the potential to attenuate adverse outcomes, making this approach a critical component of ongoing care [5].

**Psychosocial Outcomes of Childhood Cancer Survivors**

Neurocognitive late effects are among the most common psychological sequelae of childhood cancer, affecting an estimated 20–60% of survivors. Neurocognitive late effects can include difficulties with attention, processing speed, and executive functioning, which can also impact learning and memory [6]. Even when overall performance falls within normative ranges, domain-specific weaknesses are common, and up to one-third of survivors demonstrate clinically meaningful deficits [7]. Longitudinal evidence further indicates vulnerability to new-onset difficulties as cognitive and academic demands increase across childhood and adolescence [7,8].

These vulnerabilities often translate into functional impairment, including increased reliance on special education services and lower attainment of secondary and postsecondary education [9,10]. Academic disruption can also constrain participation in age-typical roles and in peer interactions, contributing to challenges in social reintegration and delays in adult milestones such as employment, financial independence, marriage, and parenthood [11,12].

Mental health difficulties often intersect with these stressors, including elevated rates of depression, anxiety, posttraumatic stress symptoms, and behavioral problems compared with siblings and population controls, as well as increased risk of suicidal ideation among higher-risk subgroups [13,14,15]. The persistence of cognitive, academic, social, and mental health challenges underscores the need for systematic surveillance and integrated psychosocial support within survivorship care [16].

**Standards of Care in Pediatric Oncology Treatment**

In response to this need, the Psychosocial Standards of Care Project for Childhood Cancer (PSCPCC), in collaboration with The Mattie Miracle Cancer Foundation, published the 15 Psychosocial Standards of Care for Children with Cancer and Their Families [17]. The standards outline an evidence-informed framework for integrating psychosocial services into routine pediatric oncology care across the treatment and survivorship continuum. Priority is placed on universal assessment at diagnosis, systematic re-evaluation at key clinical and developmental transition points, and timely access to supports including psychological and psychiatric care, neuropsychological monitoring, procedural distress management, school re-entry, and educational advocacy. Importantly, the standards extend beyond active treatment to include psychosocial components of survivorship, palliative and end-of-life care, and bereavement, emphasizing continuity and interdisciplinary coordination within pediatric oncology systems.

To operationalize these principles within survivorship clinical care, the Children’s Oncology Group (COG) developed Long-Term Follow-Up Guidelines that provide exposure-based, risk-adapted recommendations for monitoring and managing late effects among survivors of pediatric and young adult cancers [18]. Structured around specific treatment exposures, the guidelines outline screening procedures across organ-specific, neurocognitive, psychosocial, and secondary malignancy domains, while incorporating health promotion and risk-reduction strategies. Psychosocial and neurocognitive surveillance are integrated within this framework, with recommendations for screening, functional assessment, and referral when indicated. The guidelines further support survivorship care planning and position systematic, risk-based follow-up as central to comprehensive survivorship management [14,16,19].

**Traditional Models of Care in Survivorship Clinics**

In many multidisciplinary survivorship clinic models, psychosocial care is structured primarily around surveillance, with the goal of triaging identified concerns and tailoring recommendations. Although survivorship guidelines consistently emphasize psychosocial screening, fewer frameworks clearly articulate how survivorship programs should operationalize integrated psychosocial services to directly address survivors’ complex and enduring vulnerabilities, resulting in an “assessment-to-intervention” gap [18,20,21]. This gap has been attributed in part to limited clinical trial evidence to guide survivorship-specific interventions, as well as the tendency for even comprehensive guidelines to emphasize screening and referral processes more than embedded treatment pathways [21]. As a result, intervention often occurs outside of multidisciplinary survivorship clinics through referral to community providers, which can introduce access and follow-through barriers and leave unmet needs despite successful identification.

Referral-based care is often the most feasible approach given visit structure and staffing constraints and remains necessary when specialty mental health services are indicated (e.g., high-acuity symptoms, safety concerns, complex comorbidity). However, among survivors reporting psychosocial vulnerabilities that may be responsive to early intervention, referral outside the clinic may delay care and reduce follow-through. Preventive, consultative services delivered within the clinic (e.g., psychoeducation, skills coaching, and problem-solving around school/work reintegration) may help address emerging concerns more efficiently and reduce barriers to engagement [21].

At the same time, implementing integrated psychosocial intervention is challenging because survivors’ needs are broad and heterogeneous, spanning multiple behavioral health domains and levels of acuity. This complexity places substantial demands on providers and care systems [21]. Evidence-informed approaches to address these implementation challenges can be drawn from pediatric healthcare contexts where behavioral health services are routinely delivered alongside medical care, offering models that emphasize timely, low-burden intervention at the point of contact.

**Frameworks of Integrated Behavioral Health: Primary Care Services**

Integrated behavioral health in pediatric primary care is a well-established service delivery model and offers an excellent framework for closing the assessment-to-intervention gap. In pediatric primary care, embedding psychological services within routine medical care improves access, engagement, and continuity by enabling same-day consultation and brief intervention at the point of need [22,23]. Consistent with the Primary Care Behavioral Health (PCBH) model, these services typically involve brief consultations of approximately 15–30 min delivered by embedded behavioral health consultants, and growing evidence suggests that even single-session or time-limited interventions can yield meaningful clinical benefit [24,25,26]. Approaches that combine team-based care with same-day consultations allow most children to receive services immediately after a need is identified, in contrast to the delays common in non-integrated settings [24]. Similarly, same-day “warm handoffs” are associated with increased likelihood of follow-up visits [22], and integrated models in federally qualified health centers have demonstrated higher 30-day follow-up rates for conditions such as ADHD and greater overall therapy engagement [22,23].

Beyond improvements in access and engagement, systematic reviews suggest that brief consultative services, measurement-based care, and population-level strategies implemented in integrated settings are associated with improved clinical outcomes [27] and may reduce disparities by increasing screening and follow-up among underserved populations [22,28]. Consistent with these findings, studies in integrated primary care settings also demonstrate significant and sustained reductions in anxiety and depression symptoms, with some meta-analytic findings indicating comparable effect sizes to longer treatment approaches for anxiety [26,29]. Collectively, this literature supports embedding brief, consultation-based psychoeducation services within survivorship care to increase uptake of psychosocial support when needs are identified. This approach may be particularly useful for concerns appropriate for preventative, low-intensity intervention, while preserving referral pathways for higher-acuity presentations.

Although PCBH models provide a useful framework for integrating brief, consultative mental health services into medical settings, the utility of translating these models into pediatric survivorship care has not yet been studied. The inherent differences between these populations support the need for addressing this empirical gap. Indeed, survivors often experience complex, cancer-related late effects, resulting in psychosocial needs that may not parallel typical primary care presentations. Accordingly, these models are best understood as having the potential to inform psychosocial care within survivorship settings rather than offering a direct model for clinical implementation.

**The Present Study**

This study describes the process of creating an embedded trainee-led psychoeducational consultation service within an existing childhood cancer survivorship clinic and presents pilot patient satisfaction data. Building on evidence from primary care behavioral health models and recognizing the need for psychosocial consultation and intervention in survivorship settings, the current study describes the development and implementation of an embedded psychoeducation and brief-consultation service line within an existing multidisciplinary childhood cancer survivorship clinic. This service line was designed and operated by predoctoral psychology interns to provide timely, consultative psychosocial support during multidisciplinary survivorship follow-up visits, with the goal of improving access to early psychosocial education and skill building. Importantly, this service is intended to complement, rather than duplicate, existing psychosocial services provided by social work and (neuro)psychology visits. Social work services often emphasize care coordination, resource provision, and broader psychosocial support. Psychologists often emphasize screening and triaging of mental health, neurocognitive, and academic concerns with a focus on providing recommendations and making referrals. Relatively less emphasis is placed on direct psychoeducation and consultation in response to identified areas of challenge or need. The present service model focused on brief, targeted mental health consultation and psychoeducation within a limited, single-session timeframe.

In this paper, we describe the structure of our survivorship clinic, the rationale and guiding framework for the embedded service model, and the operational features of the service line. We also present pilot data on patient reception and satisfaction with the novel service line within routine survivorship care. By detailing this service model and presenting initial patient feedback, we aim to illustrate how survivorship clinics can expand beyond primarily assessing psychosocial needs to incorporate more immediate and direct clinic-based support. Furthermore, this example highlights the educational and clinical benefits of psychology trainees operating this service line and, in turn, the practicality and scalability of incorporating similar embedded models into existing survivorship programs.

## 2. Methods

**Pre-existing Clinic Model**

The Doernbecher Children’s Hospital survivorship clinic serves survivors from a three-state region (Oregon, Southwest Washington and far Northern California) who have been impacted by oncological, hematological, or immunological disorders in childhood and are at least two years post-treatment completion and in remission. We see patients of all ages, as there is currently no well-established option for transition to adult care in our area. Team members include a physician or advanced practice provider, neuropsychologist, social worker, dentist, registered nurse, and research associate. Prior treatments include chemotherapy, radiation, bone marrow transplant, surgery, and other emerging interventions. Patients are referred by their primary care providers or hematology/oncology specialists. The clinic primarily operates in person; however, virtual telehealth options are available to meet the needs of our rural community members when appropriate [30].

The clinic sees up to seven patients in an afternoon. Most patients meet all disciplines during a single visit, although schedules are modified occasionally based on individual needs. Patients remain in one exam room for the duration of the appointment, with each team member entering sequentially. Each discipline focuses on screening for late effects associated with childhood cancer treatment as well as education on health promotion and disease prevention. The breadth of disciplines allows for comprehensive surveillance across medical, neurocognitive, psychosocial, and functional domains within a biopsychosocial framework.

**Development of a Novel Service Line**

The survivorship clinic expanded its services by adding a psychology intern to provide consultative and brief psychoeducation-focused mental health services, complementing existing neuropsychological and social work screening, triaging, and referral processes. Psychology interns are doctoral trainees who have completed their didactic coursework (typically 4–5 years) and multiple years of supervised clinical practica. The predoctoral clinical internship represents the final year of required clinical training prior to earning their doctoral degree and pursuing licensure as a psychologist. Within the state of Oregon and our institution, predoctoral clinical psychology interns may provide independent psychoeducational-focused consultations to patients under the supervision of a licensed psychologist, without the need for live supervision during each encounter. Given the consultative focus of the service, billing codes restricted to independently licensed psychologists are not required. Further, embedding this service within the existing multidisciplinary clinic infrastructure does not impose substantial additional demands on clinical space or scheduling.

**Evaluation of Needs.** The first step toward developing the new service line involved identifying the types of services that would be most valuable to the clinic’s patients. The intern began by learning about the clinic structure and conducting qualitative observation of common behavioral, emotional, and cognitive/academic concerns identified during patient visits with the neuropsychologist and social worker.

Next, the intern interviewed clinic faculty and staff to ascertain their perceptions of unmet patient psychosocial needs. From these discussions, it became clear that many patients were struggling with common mental health, behavioral, social, and school-related concerns, many of which did not rise to the level warranting a formal mental health diagnosis or long-term intervention. Though consistent with findings in the extant literature [6,7,9,10,14,15], it was not uncommon for patients to endorse clinically significant neurocognitive or psychosocial concerns, often related to their cancer history.

Under the existing model, the neuropsychologist and social worker routinely assessed mental health needs and provided psychoeducation to build motivation for treatment engagement; however, time constraints often limited the depth of this intervention, resulting in a heavy reliance on referrals to community-based providers. In the Portland metropolitan area, mental health services often have long waitlists, particularly for children and adolescents, resulting in delays of several months or longer before treatment initiation. These systemic barriers further impede timely access to needed care.

Finally, a needs assessment was conducted based on a literature review of the most common emotional, behavioral, cognitive, and academic outcomes among childhood cancer survivors. The literature identified similar themes consistent with clinical observation and staff interviews. Specifically, a multimodal needs assessment highlighted that patients with a history of childhood cancer are more susceptible to neurocognitive vulnerabilities, academic disruption, and co-occurring mental health difficulties that may evolve or intensify as developmental demands increase [6,7,9,10,14,15]. Overall, common needs identified included stress, anxiety, mood, sleep difficulties, behavioral issues, cognitive difficulties, and securing school services.

**Working Within Constraints.** Several structural constraints influenced the development of the service line. The first major constraint was that psychology interns at our institution cannot provide or independently bill insurance for professional psychological services without live supervision from a licensed psychologist. However, psychoeducation-focused consultative services do not require independent psychology licensure within the state of Oregon or under our institutional policies. These factors informed the decision to design the service line as brief, integrated within the existing clinic, and psychoeducational in scope. The decision was further supported by prior research on integrated primary care models demonstrating that the introduction to and delivery of brief, individually tailored psychological consultation increases rates of patient engagement and follow-up with long-term mental health services [22,23].

**Service Description.** The intern and supervising psychologist developed a 20-min consultative service to be incorporated into patient visits as indicated. The service was designed to provide brief psychoeducation and skills targeting common psychosocial and behavioral concerns. The service complemented the existing neuropsychology and social work visits; the existing visits focused on assessing needs or concerns, whereas the intern-led consultation service placed greater emphasis on education and skill-building. Content was organized into modular topics that could be delivered in the allotted time frame. Modules included improving mood, general coping with stress, managing anxiety, parent-focused strategies for responding to common childhood behavioral concerns, sleep hygiene, enhancing time management and task initiation, and navigating formal school-based accommodations and supports. Across implementation of the service, additional needs were identified, including managing chronic pain and parent–child collaboration; however, data were not collected on these topic areas as they were created following data completion. For patients presenting with clinically significant mental health concerns, consultation also focused on introducing the nature of empirically supported psychotherapies relevant to the identified concerns. Several modules (e.g., task initiation and time management, parent-focused strategies for responding to problematic behavior) were developed and implemented during the first year of service implementation. At the initiation of the service (October 2023), modules included general coping with stress, managing anxiety, improving mood, sleep hygiene, and accessing school support. In January 2024, the module on parent responses to problematic behavior was established. In April 2024, the module on task initiation was established. Modules on pain management and collaborative problem solving were established in the fall of 2024, following the end of pilot data collection. Each consultation followed a consistent structure: an introduction to the service, brief information-gathering to tailor recommendations, general psychoeducation regarding the concern, and targeted skills training. Handouts were developed for each module, summarizing key psychoeducational content and empirically- or systems-based skills and strategies for families to use at home. Handouts went through institutional review for health literacy, were branded and approved, and were translated into Spanish.

**Figure 1:**
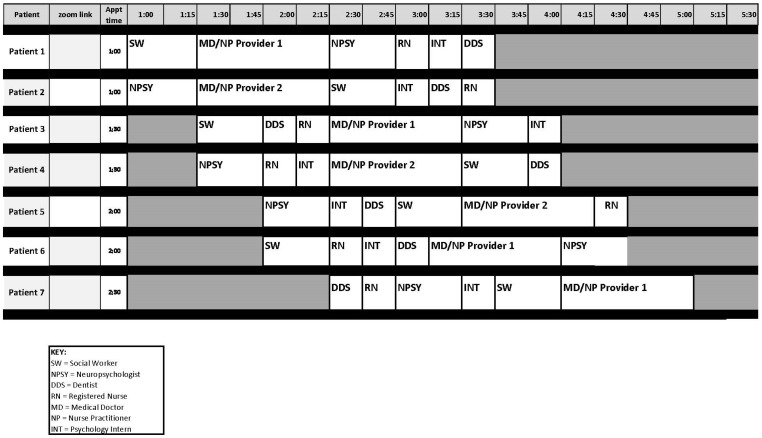
Visual representation of clinic structure and patient flow. Patients meet with each discipline for individual visits. SW and NP visit results inform focus of psychology intern’s psychoeducational consultation visit.

The psychological consultation service was designed to follow the social work or neuropsychology visit so that the focus could be identified in advance. Specifically, the neuropsychologist or social worker would review patients with the psychology intern ahead of the intern’s visit and would help identify which module was most appropriate for each patient. See Figure 1 for a visual overview of clinic structure and patient visit flow across the various disciplines. Services were optional and could be declined at any time. If multiple concerns were identified, the patient and family would select the primary concern to prioritize during the consultation. Time permitting, the intern covered multiple topic areas if the neuropsychologist, social worker, and/or patient expressed wanting support for more than one challenge area. As mentioned, because the service was consultative and educational in scope rather than providing tailored psychotherapy, families were not billed for the service.

**Implementation.** Following a two-month development phase, the intern began delivering the consultative service model within the clinic (in October 2023). One intern was involved with the initial development of the service and was the only intern to provide the service for the first nine months of its existence and the data collection period. A second intern continued the service into its second year and helped develop additional modules (i.e., pain management and collaborative problem solving) that were incorporated into the service.

**Evaluation of Service**

At the conclusion of each consultation, a brief digital satisfaction survey was administered to assess service receptivity and satisfaction. The survey was developed internally and was not validated. The survey was only offered in English. The questionnaire was delivered at the conclusion of the visit. The survey was administered via web browser on the clinic room computer. The intern introduced the survey at the end of the service, invited families to complete it, and then exited the room to allow privacy. Older adolescents and adult survivors typically completed the survey independently, whereas parents most often completed it on behalf of younger children. The subsequent provider closed the web browser at the start of the next visit, once the survey had been completed. Identifiable patient data were not collected as part of the questionnaire and were thus not linked to patient responses. As the study did not collect identifiable data and focused primarily on service development, institutional review board review was not required.

The survey was developed to assess the primary topics addressed, perceived helpfulness, and appropriateness of the visit length. Families were asked to select which module(s) were discussed from a predefined list, with the option to endorse multiple topics. They also rated overall helpfulness on a 5-point Likert scale, with the following response options: very helpful, helpful, neither helpful nor unhelpful, unhelpful, or very unhelpful. Additionally, families were asked if the time spent providing the service felt appropriate or whether more or less time would have been preferred. Finally, respondents were presented with an open-ended prompt to share additional written feedback (see Supplementary S1 for a copy of the questionnaire). Survey data collection occurred over an eight-month period beginning in November 2023 (approximately one month into the initiation of the service) and ending in June 2024. Given that the pain management and collaborative problem-solving modules were not created within that time frame, patient satisfaction data and frequency data were not collected as related to those modules. The survey was not updated across the service provision, with the exception of adding new modules to the topic list when they were implemented.

## 3. Results

Data were collected over eight months during the intern’s first year of service provision (November 2023–June 2024). During this period, 74 of the 102 patients seen in clinic (73%) received the novel consultation service. Instances in which patients did not receive the service were due to patient or clinic time constraints or the visit was declined. Specifically, only five patients (6%) of patients offered the service chose to decline the service. Of the 74 patients seen, 48 patients (65%) completed the satisfaction survey. Given the absence of corresponding demographic or clinical data, analyses comparing questionnaire responders and nonresponders could not be conducted. Survey responses were analyzed using descriptive statistics regarding perceived utility and satisfaction with the length of the visit. Additionally, responses were analyzed using frequency distributions of the topics discussed during the visit.

**Patient Satisfaction**

Overall, respondents reported high levels of satisfaction with the service. When asked to rate helpfulness, 90% (*n* = 43) indicated that the service was either “Helpful” or “Very Helpful.” One respondent rated the service as “Neither Helpful nor Unhelpful.” Two respondents (4%) rated the service as “Unhelpful,” and another two (4%) rated it as “Very Unhelpful.” Respondents were also generally satisfied with the duration of the consultation. Nearly 90 percent (*n* = 43) indicated that the time allotted felt appropriate. A small percentage (8.33%, *n* = 4) expressed a desire for additional time, and only one respondent (2.08%) preferred less time.

We did not employ a qualitative analytic strategy to review open-ended feedback and, thus, those data are not presented here.

**Frequency of Topics Discussed**

Frequency data was not available for two modules (i.e., pain management and collaborative problem solving). Of the remaining modules, thirty-one participants (65%) indicated that more than one concern was addressed during the consultation. Of those who endorsed discussing multiple topics, 26 reported discussing anxiety (81%), indicating anxiety is often comorbid with other psychosocial concerns. Overall, anxiety management was the most frequently discussed topic, endorsed by 69% of respondents (*n* = 33). General coping with stress was the second most frequent topic, identified by 50% (*n* = 24) of respondents. Acquisition of school-based services (38%, *n* = 18) and sleep hygiene (31%, *n* = 15) were the next most frequently endorsed topics. Parent-focused training for behavioral concerns and mood management was endorsed by 17% of respondents (*n* = 8) each. Task initiation was the least frequently discussed topic, endorsed by 8% (*n* = 4). However, frequency data for parent-focused training for behavioral concerns and task initiation modules were introduced later into data collection, thereby skewing frequency data.

Analysis of frequency data was repeated for two separate time windows. Specifically, frequency data were analyzed from January 2024 (when the parent-focused module was implemented) through the end of data collection (June 2024). During this time window, 35 patient responses were collected. Anxiety remained the most commonly discussed topic (35%), followed by sleep hygiene (18%). Coping with stress, accessing school-based services, and parent responses to challenging behavior shared the same frequency data and were each discussed in 15% of patient visits. Only one visit focused on improving mood during this time window.

Additionally, frequency data were analyzed from April 2024 (when the task initiation module was implemented) through the end of the data collection period. Twelve patient responses were collected during this time window. Frequency data were as follows: anxiety management (21%), accessing school services (19%), coping with stress (19%), sleep hygiene (17%), task initiation (12%), parent responses to challenging behavior (12%), and mood management (0%).

## 4. Discussion

The current paper describes the development and implementation of a novel trainee-led psychoeducational and consultative service delivered within an established childhood cancer survivorship clinic at a tertiary academic medical center in the Pacific Northwest of the United States. Within this new framework, a predoctoral psychology intern delivers tailored consultations lasting approximately 20 min during a survivorship clinic appointment. Consultation modules include coping with general stress, anxiety management, low-mood support, time management and task initiation skills, parent-focused strategies for behavioral concerns, sleep hygiene, and navigating formal school-based supports. Modules implemented after the service’s first year include pain management and an introduction to collaborative problem-solving. Pilot patient satisfaction data indicate that the majority of families (nearly 90%) found the service helpful. Most respondents reported that the consultation duration was appropriate, with fewer than 10% expressing a desire for more or less time with the psychology intern. The most discussed topic was anxiety management.

The creation of an integrated psychological consultation service within survivorship programs addresses several existing care gaps. Providing brief consultation within an existing medical visit can effectively address many patients’ initial concerns [22,23,24,25,27]. Brief interventions provided within integrated medical care settings may further help to reduce disparities in mental health service access for historically underserved populations [22,28], particularly when offered virtually [30]. However, given the absence of patient demographic data, the potential for reducing disparities within the current patient population remains speculative and thus represents an avenue for future research. Further, embedding these services within existing medical visits minimizes additional time burden while increasing familiarity with, and access to, mental health services that might otherwise feel less familiar and/or inaccessible.

The utilization of psychology trainees to deliver this service enhances patients’ access to behavioral health services. Within the state of Oregon and at our institution, the psychoeducational and consultative nature of the service does not require live supervision from a licensed psychologist or the use of professional psychology billing codes. Thus, mental health visits can be conducted with greater capacity, as simultaneous supervision by a psychologist is not required. Moreover, without an embedded billing structure, patients can access these services without additional insurance or financial burden. Embedding this service within an existing survivorship clinic model minimizes the need for additional clinical space and scheduling resources. Patients are already scheduled to meet with the multidisciplinary team, allowing brief consultation to be incorporated without requiring separate appointments or additional scheduling resources. Additionally, within our program, patients remain in a single clinic room while providers rotate to see patients and families individually. Thus, integrating a brief consultative service simply extends the use of the existing room for a short period without necessitating additional clinical space.

Beyond enhancing patient care, the opportunity for interns to develop and independently deliver consultative services aligns with key psychology training initiatives and standards. During the predoctoral internship period, trainees are tasked with increasing autonomy in clinical decision-making and service delivery. Further, the American Psychological Association’s (APA’s) Standards of Accreditation emphasize psychological consultation and multidisciplinary collaboration as a core training competency prior to licensure [31]. Finally, the development and evaluation of the service line emphasize program evaluation and quality improvement skills, which are key elements of professional psychology careers [32].

The current study is not without limitations. First, evaluation of service implementation and patient satisfaction relied on optional participation in a post-visit survey and used a single-item for the outcome measures without formal measure validation. This approach enabled access to a unique patient population, flexibility in service development, and rapid incorporation of patient satisfaction. However, in doing so, we are unable to report on patient demographic and clinical data, limiting the ability to assess the generalizability of the pilot findings. Relatedly, the lack of demographic or clinical data describing the current sample precludes the generalization of the current findings to larger patient populations. Consequently, the current results present patient satisfaction data limited to this sample only. Additionally, although no identifiable patient data were collected, response biases (e.g., social desirability, optional survey completion) may be present, potentially overestimating perceived helpfulness and failing to capture negative perceptions of the service. The current methodological approach also lacks a direct comparison group, thereby limiting the ability to compare the outcomes of the novel service with standard survivorship care or alternative models (e.g., integrated consultation models within pediatric primary care). As described in the methodology section, service modules continued to be developed throughout service implementation and data collection. Analysis of frequency data relies on smaller sample sizes for modules introduced mid-data collection (i.e., parent responses to problematic behavior, task initiation), and no frequency data are available for the pain management and collaborative problem-solving modules, which were developed after data collection was complete. We also recognize that some benefits of the current model may depend on state regulations and institutional training structures. In particular, variability in state and/or institution restrictions on service delivery by predoctoral interns practicing without live supervision, as well as differences in billing eligibility, may limit generalizability across settings. Finally, the current service, in its entirety, was available in English and Spanish only, and patient satisfaction data were obtained only among those with English proficiency, thereby limiting generalizability to other linguistic populations.

Several important avenues for future research emerge from this work. First, the current model requires investigation to determine how patient demographic and clinical data are associated with satisfaction and frequency data. Moreover, investigations using validated psychosocial and neurocognitive outcome measures are a crucial next step for understanding the efficacy of the service as it relates to mental health and cognitive functioning. There is a pressing need to understand the extent to which participation in integrated consultation models within survivorship care is associated with downstream outcomes as well. Specifically, future research should evaluate whether service engagement results in measurable improvements in outcomes over time and whether engagement in the service enhances subsequent participation in mental health services. While an integrated consultative model has potential to minimize the assessment-to-intervention care gap, further work is needed to demonstrate this phenomenon within a survivorship population. Comparison of the current model with survivorship standard-of-care models will aid in ascertaining the additive benefit of this novel service line. Relatedly, comparing the current model with integrated consultation services in pediatric primary care may illuminate whether implementing such services in survivorship settings yields comparable benefits. Additional research is also needed to investigate whether the current service functions similarly across both majority and historically marginalized communities. Although the integrated model may help address care gaps, it remains unclear whether patients from diverse ethnographic and socioeconomic backgrounds demonstrate comparable levels of receptivity and benefit.

## 5. Conclusions

The current paper highlights the process of creating an innovative, trainee-led psychoeducational consultative service within an existing childhood cancer survivorship program. Pilot patient satisfaction data demonstrate that the service is perceived to be helpful and that the length of the service is adequate. Advantages include providing a no-fee service that places minimal burden on clinic operations (e.g., scheduling, clinic space), as well as rapid access to mental health services with a cancer-informed clinician, particularly in the context of long waitlists for community-based care. Additionally, the program model contributes to the advancement of psychology training, including program evaluation and development, as well as opportunities for autonomous service delivery. Future directions include replication of service implementation using validated outcome measures, examination of long-term benefits of service provision, and comparison of the current model with standard-of-care survivorship programs and with similar models used in other settings, such as pediatric primary care.

## Data Availability

Please contact the corresponding author (MV) for access to data used within the current study.

## Supplementary Materials

The following supporting information can be downloaded at: https://www.mdpi.com/article/10.3390/children13050656/s1, Internally developed patient satisfaction questionnaire.
