# Supplementary material for: Novel Trainee-Led Psychological Service in Childhood Cancer Survivorship Clinic: A Process Paper
Source: Children (Basel). 2026 May 7;13(5):656. doi: 10.3390/children13050656 (PMC13204786; doi:10.3390/children13050656)
Supplement: Supplementary file 1 [file children-13-00656-s001.zip › children-4213314-supplementary.pdf]

Supplement S1. Internally developed patient satisfaction questionnaire.

Thank you for taking the time to complete this survey. Please answer the questions on the following page as they relate to your visit with the psychology intern in the survivorship clinic. This is a new service we are offering, and your honest feedback will help us meet your needs in future visits.

How helpful was your conversation with the psychology intern?

- o Very Helpful
- o Unhelpful
- o Neither helpful nor unhelpful
- o Helpful
- o Very helpful

Do you feel the time spent with the psychology intern was the correct amount for your needs?

- o No, I would have preferred less time
- o No, I would have preferred more time
- o Yes, that felt like the right amount of time

What did the psychology intern speak to you about today (can select multiple topics)?

- o Anxiety
- o Behavior Problems
- o Depression
- o School
- o Sleep
- o Stress
- o Task Initiation

Please add any other feedback you have here:

